# HIST1H4L promotes small cell lung cancer tumorigenesis by regulating the PI3K/Akt pathway and serves as a potential diagnostic biomarker

**DOI:** 10.3389/fonc.2026.1959848

**Published:** 2026-09-14

**Authors:** Shicheng Feng, Min Feng, Zhiqiang Lu, Jie Shen, Ying Xia

**Affiliations:** 1 Radiotherapy Department, Zhongda Hospital, Southeast University, Nanjing, China; 2 Radiology Department, The Affiliated Brain Hospital of Nanjing Medical University [Chest Area], Nanjing, China; 3 Department of Hematology & Oncology, Jurong Hospital Affiliated to Jiangsu University, Zhenjiang, China

**Keywords:** biomarker, cancer, differentially expressed genes, prognosis, small cell lung cancer

## Abstract

**Objective:**

Despite advances in treatment, small cell lung cancer (SCLC) remains highly aggressive and prone to relapse, leading to poor patient outcomes. Histone HIST1H4L is a key protein that maintains chromatin structure, but its role in SCLC is still unclear. This study aims to define its oncogenic role in SCLC and to determine whether it drives malignant progression by activating the PI3K/AKT signaling pathway.

**Materials and methods:**

Public datasets were used to integrate HIST1H4L expression with differentially expressed genes. Functional bioinformatics investigations and survival association analyses were then conducted. Additionally, stable HIST1H4L knockdown cell lines were generated. Knockdown efficiency was confirmed at the mRNA and protein levels by qRT-PCR and Western blot (WB). Cell proliferation was measured using CCK-8 and EdU staining. Cell invasion was assessed with Transwell assays. Cellular senescence and apoptosis were evaluated by β-galactosidase staining and flow cytometry, respectively. Finally, WB was used to detect changes in key proteins of the PI3K/AKT signaling pathway after HIST1H4L knockdown.

**Results:**

HIST1H4L was significantly upregulated in SCLC tumors versus normal tissues and enriched in DNA transcription-related pathways. Critically, high HIST1H4L expression correlated with poor patient survival. HIST1H4L knockdown markedly suppressed proliferation, migration, and invasion. It also reduced senescence and apoptosis. HIST1H4L knockdown weakened the activation of the PI3K/AKT signaling pathway. The levels of p-AKT and p-PI3K decreased, while total AKT and PI3K levels remained unchanged.

**Conclusion:**

HIST1H4L is significantly overexpressed in SCLC and correlates with poor prognosis. It functions as a key oncogenic driver by activating the PI3K/AKT pathway, promoting tumor proliferation and invasion.

## Introduction

Small cell lung cancer (SCLC) is an aggressive neuroendocrine malignancy closely linked to tobacco exposure. It accounts for about 15% of all lung cancer cases, with roughly 150,000 new cases worldwide each year (1). For decades, SCLC has remained a major challenge in thoracic oncology, with little progress in treatment. SCLC development is driven by complex mechanisms. These include gene mutations, protein inactivation, and changes in the tumor microenvironment. At diagnosis, about 70% of patients already have distant metastases and are in the extensive stage, while only 30% are in the limited stage (2). The prognosis is poor. The 5-year survival rate is only about 7% (3). This is mainly due to late diagnosis and the highly aggressive nature of the disease. Although SCLC is initially sensitive to chemotherapy, almost all patients eventually develop resistance. This leads to disease progression and limits long-term benefit. These findings highlight the ongoing burden of SCLC and the urgent need for new strategies in early diagnosis and treatment. To move beyond a one-size-fits-all approach, more precise targeted therapies are needed. This requires identifying specific biomarkers that are highly expressed in SCLC tumor cells but show minimal expression in normal tissues. In-depth exploration of the signaling pathways that drive SCLC and the development of corresponding novel targeted therapies are crucial for improving patient prognosis. However, the transition from laboratory discoveries to clinical applications faces both opportunities and challenges, and cross-disciplinary collaboration and systematic research are urgently needed (4).

Histone-associated genomic variations are commonly observed across diverse cancer types. Such alterations can disrupt nucleosome stability, post-translational modifications of histones, and DNA repair mechanisms, thereby promoting the expression of oncogenes (5, 6). Studies have reported that approximately 6% of patients harbor histone H4 gene mutations, with over 50% of non-melanoma skin cancer patients exhibiting such alterations. Histone H4 mutations have also been identified in tobacco-related tumors, including lung cancer (6). Among histones, HIST1H4L (also known as H4C13) is a core member of the H4 gene family. As an essential component of the nucleosome, the basic unit of chromatin, it plays a key role in maintaining chromosomal stability. Notably, the function of HIST1H4L is highly tissue- and disease-specific. Previous studies have revealed its complex regulatory roles in multiple cancers. In lung adenocarcinoma, HIST1H4L dysregulation is closely linked to cellular senescence and may act as a senescence-associated gene (7, 8). In prostate cancer with ERG fusion, HIST1H4L expression is specifically upregulated and is considered a potential downstream target of the ERG fusion, although its precise oncogenic mechanism remains unclear (9, 10). Additionally, HIST1H4L has been identified as a key regulator of malignant phenotypes in esophageal and lung cancers, and its abnormal expression may drive tumor progression (11).

Recent studies suggest that HIST1H4L may be involved in the regulation of SCLC and may be associated with patient prognosis (12). This finding offers new insight into the epigenetic mechanisms of SCLC. However, although its roles in other cancers have been reported, the specific biological functions of HIST1H4L in SCLC and the underlying molecular mechanisms remain unclear. Given its importance in tumor regulation and its potential link to SCLC, this study aims to systematically investigate the biological roles of HIST1H4L in SCLC cells and clarify its mechanism of action. The goal is to better understand SCLC pathogenesis and to identify new potential therapeutic targets.

## Methods

### Data collection and processing

SCLC-related miRNA microarray data from the GSE60052, GSE30219 and GSE149507 datasets were downloaded from the public Gene Expression Omnibus (GEO) database (https://www.ncbi.nlm.nih.gov/geo/). A threshold of *P* < 0.05 and |logFC| > 2 was applied to identify significantly differentially expressed genes, where fold change (FC) represented the magnitude of differential expression. The target gene HIST1H4L was visualized using a volcano plot. Additionally, Protein–Protein Interactions (PPIs) were predicted using the Search Tool for the Retrieval of Interacting Genes/Proteins (STRING) database (https://string-db.org/) (13) and visualized using Cytoscape software (version 3.10.4, https://cytoscape.org/). respectively. Functional enrichment analysis was also conducted on the STRING platform.

### Validation of HIST1H4L expression and its potential role in SCLC

This exploratory retrospective study included 91 consecutive patients diagnosed with SCLC between December 2010 and April 2022 at our institution. Tumor specimens were obtained through core needle biopsy, endobronchial biopsy, or surgical tissue resection. The inclusion criteria were as follows: (1) histologically confirmed SCLC; (2) complete clinical and medical records; (3) age ≥ 18 years; (4) available TNM staging information; and (5) no history of preoperative adjuvant treatments, such as chemotherapy or radiotherapy. Exclusion criteria included: (1) unclear pathological subtype; (2) coexistence of other malignancies; (3) loss to follow-up; (4) incomplete clinical information; and (5) presence of severe hepatic, renal, or cardiovascular diseases. Eligible patients were followed to record survival status and duration. The follow-up endpoint was March 2022. Overall survival (OS) was defined as the interval from the initial diagnosis to death or the last follow-up. The research was conducted with ethical clearance granted by the Institutional Ethics Committee of our hospital (approval number: 2022ZDSYLL252-P01).

### Immunohistochemistry analysis of clinical samples

Immunohistochemistry was performed to evaluate HIST1H4L protein expression in SCLC tissues. Paraffin-embedded tissue blocks were sectioned at a thickness of 4 μm. Sections were fixed in 4% paraformaldehyde, embedded in paraffin, deparaffinized in xylene, and rehydrated through a graded ethanol series. Antigen retrieval was conducted using sodium citrate or buffer. To prevent nonspecific binding, sections were blocked with 10% sheep serum in phosphate-buffered saline (PBS). Slides were incubated with primary antibodies against HIST1H4L overnight at 4 °C. After washing with PBS, biotin-labeled secondary antibodies and streptavidin-horseradish peroxidase were applied and incubated at room temperature for 10 minutes each. Visualization was achieved using 3,3’-Diaminobenzidine, followed by dehydration and resin mounting. Immunoreactivity scoring was performed using a standard semiquantitative system, in which the staining index was calculated as the product of the percentage of HIST1H4L-positive cells and staining intensity.

### Cell lines and culture conditions

Human SCLC cell lines (H446, H146, and H526) and a normal human lung epithelial cell line (BEAS-2B) were obtained from the Shanghai Cell Bank, Chinese Academy of Sciences. Cells were cultured in Dulbecco’s Modified Eagle Medium supplemented with 10% heat-inactivated fetal bovine serum, 100 μg/mL penicillin, and 100 μg/mL streptomycin. Cultures were maintained at 37 °C in a humidified incubator with 5% CO_2_.

### Lentiviral transduction

The HIST1H4L mRNA sequence was retrieved from the NCBI database to design three different shRNAs targeting HIST1H4L. The most effective shRNA was selected for subsequent experiments. Lentiviral vectors used were based on GV493, with the original sequence: hU6-MCS-CBh-gcGFP-IRES-Puro. The control sequence was TTCTCCGAACGTGTCACGT. The sh-HIST1H4L target sequences (5′-3′) were: CAGGCCTTATATACGAGGAGA; TGGCACGGCGTGGAGGCGTTA; AGTTCTGCGCGACAACATTCA. Cells were transduced with the lentiviral particles and cultured in medium containing 10 μg/mL puromycin. Among the three stable cell lines generated, the one showing the highest knockdown efficiency was used for the functional assays.

### Quantitative real-time polymerase chain reaction

qRT-PCR was used to measure HIST1H4L mRNA levels in cells. Total RNA was extracted using Trizol reagent and reverse-transcribed into cDNA according to the manufacturer’s instructions. PCR amplification was performed using SYBR Green dye. The reaction mixture contained cDNA template, primers, SYBR Green Master Mix, and nuclease-free water. Cycling conditions were: 95 °C for 30 s pre-denaturation, followed by 40 cycles of 95 °C for 5 s and 60 °C for 30 s. GAPDH was used as the internal control, and relative expression levels were calculated using the 2^−ΔΔCt method.

### Western blot analysis

Proteins were extracted using RIPA buffer (Beyotime), separated on 10% SDS-PAGE gels, and transferred to polyvinylidene fluoride membranes. The membranes were blocked with 5% non-fat milk for 1 hour at room temperature. They were then incubated with primary antibodies overnight at 4 °C, followed by incubation with secondary antibody at room temperature for 1 hour. Protein signals were detected using ECL reagent (Beyotime) and quantified with ImageJ software.Primary antibodies used included: anti-HIST1H4L (ab109463, 1:1000; Abcam), anti-PI3K (ab140347, 1:2000; Abcam), anti-p-PI3K (BS4605, 1:1000; Bioworld), anti-Akt (1:500; Abcam), anti-p-Akt (1:500; Abcam), and anti-GAPDH (ab181602, 1:5000; Abcam). The secondary antibody was goat anti-rabbit IgG (BL003A, 1:4000; BioSharp).

### Cell counting kit-8 assay

H146 cells were counted and seeded into 96-well plates, with three replicates per group. The control group had the culture medium refreshed regularly. At 12 h, 24 h, 48 h, and 72 h, 10% CCK-8 solution was added to the medium. Absorbance at 450 nm was measured using a microplate reader to assess cell viability.

### EdU assay

Cell proliferation was assessed by measuring DNA synthesis using the Cell-Light™ EdU Apollo^®^567 Cell Tracking Kit (RiboBio). SCLC cells were seeded in 48-well plates and cultured for 2 days. Cells were then pre-treated for 48 h, followed by EdU incubation for another 48 h. The number of EdU-positive cells was recorded under a fluorescence microscope.

### Transwell assays

Transwell assays were performed to assess cell migration capacity. Specifically, cells were transfected with different plasmids and then inoculated into the top compartment with serum-free medium, while medium containing 10% FBS was added to the lower compartment. After 48 h of incubation, the remaining cells in the top compartment were removed with a cotton swab, and the cells in the lower compartment were stained with 0.1% crystal violet for 10 min. Finally, the invasion cells were measured and photographed under a microscope.

### β-Galactosidase staining

β-Galactosidase staining was performed following the manufacturer’s instructions. Cells were washed with PBS and fixed with β-galactosidase fixative at room temperature for 15–20 minutes. After washing with PBS, an appropriate amount of staining solution was added, and cells were incubated overnight at 37 °C. Positive cells were identified by blue staining under a light microscope, photographed, and recorded.

### Flow cytometry

After treatments, 1 × 10^6^ cells from each group were collected for apoptosis analysis. Cells were stained with 5 µL Annexin V and 10 µL propidium iodide in 500 µL binding buffer for 15 minutes at room temperature in the dark. Apoptosis was then analyzed using flow cytometry and quantified with FlowJo software (version 10.5.0).

### Statistical analysis

Data were analyzed using SPSS 19.0 or R software (version 4.4.1, http://www.R-project.org). Unless otherwise specified, quantitative data are presented as mean ± SD. Continuous variables were analyzed using Student’s t-test, and categorical variables were compared using Pearson’s chi-square test. Differences in OS were assessed using Kaplan-Meier (KM) survival analysis. A *p*-value < 0.05 was considered statistically significant.

## Results

### HIST1H4L expression in SCLC based on GEO datasets

Analysis of the GSE60052 dataset showed that HIST1H4L expression was significantly upregulated in SCLC samples compared with normal controls (**Figure 1A**). This finding was further validated using two independent datasets, GSE30219 and GSE149507. In both datasets, HIST1H4L expression was markedly elevated in SCLC tissues relative to normal lung tissues (*P* < 0.05) (**Figures 1B, C**).

**Figure 1:**
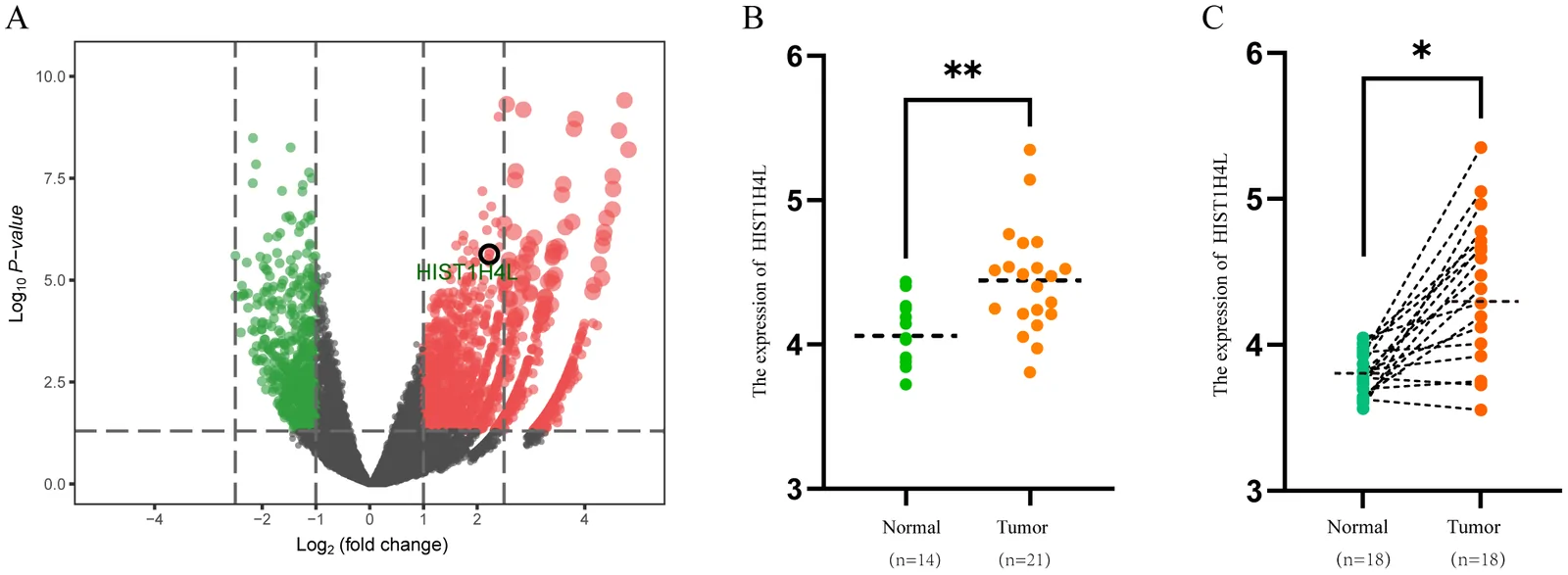
Differential expression analysis of HIST1H4L in SCLC patients. **(A)** Volcano plot of HIST1H4L based on the GSE60052 dataset. Upregulated differentially expressed genes are shown in red, while downregulated genes are shown in blue. **(B)** the GSE30219 dataset. **(C)** the GSE149507 dataset. (*P < 0.05; **P < 0.001).

### PPI network construction and functional enrichment analysis of HIST1H4L

STRING-based PPI network analysis identified 10 key interacting partners for HIST1H4L, including H2AC8, H3C12, H3C13, H3-4, H3-3B, KMT5A, CENPA, H2AX, EP300, and PRMT5 (**Figure 2A**), which was then visualized in Cytoscape. Kyoto Encyclopedia of Genes and Genomes (KEGG) pathway analysis (**Figure 2B**) implicated HIST1H4L in several disease-related pathways, including “Transcriptional misregulation in cancer,” “Systemic lupus erythematosus,” and “Alcoholism.” Gene Ontology (GO) analysis further revealed significant enrichments across three domains (**Figures 2C–E**). In Molecular Function (MF), the top terms were “Structural constituent of chromatin,” “DNA binding,” and “Protein binding.” For Biological Process (BP), enrichment was centered on “Nucleosome assembly,” “Chromatin remodeling,” and “Protein localization to chromosome.” Finally, Cellular Component (CC) analysis highlighted that these genes are primarily localized to “Nucleosome,” “Chromatin,” and “Chromosome.”

**Figure 2:**
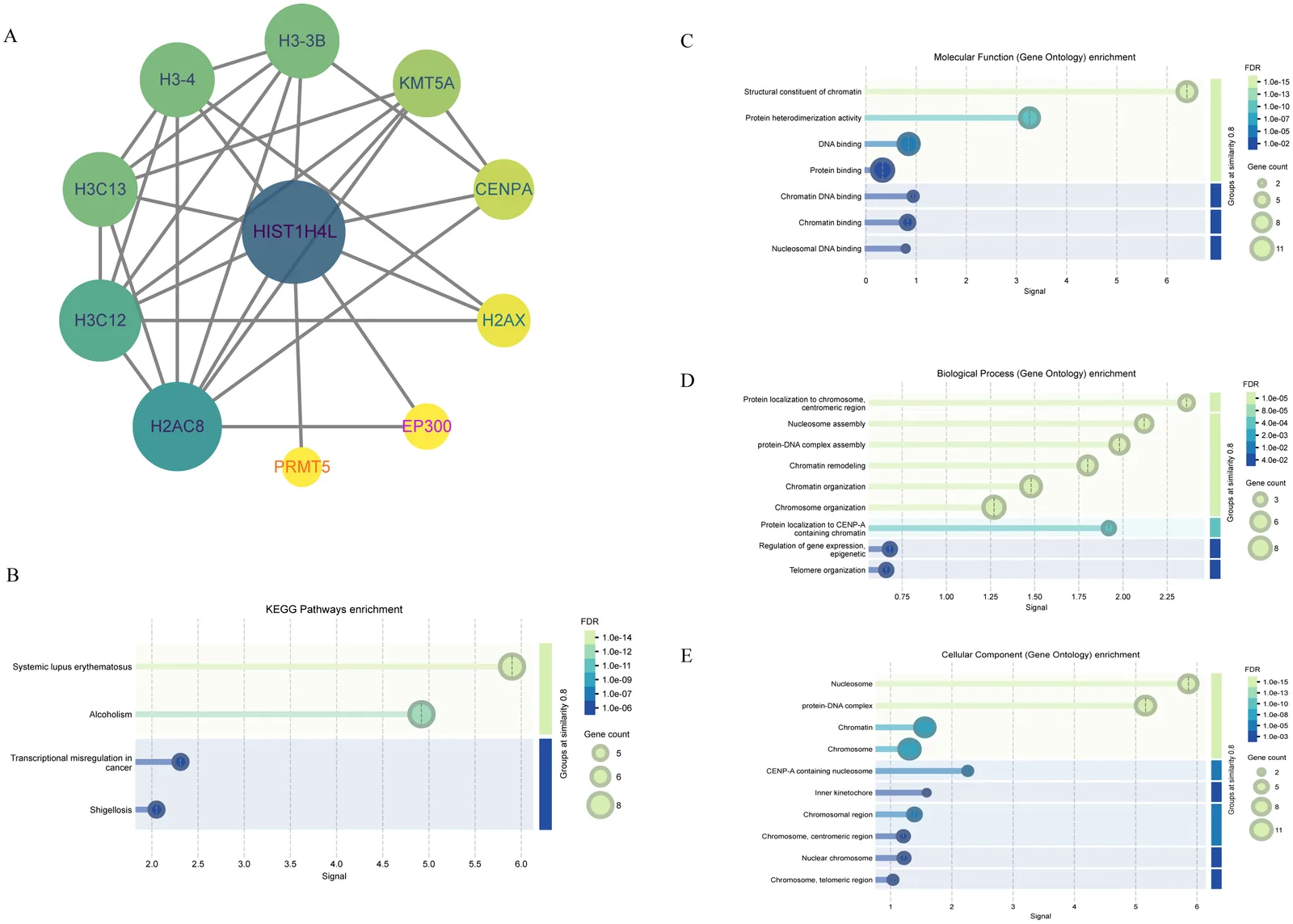
Protein-protein interaction (PPI) network and functional enrichment analysis of HIST1H4L-related genes. **(A)** PPI network. **(B)** KEGG pathway enrichment analysis. **(C)** molecular function (MF) enrichment analysis. **(D)** biological process (BP) enrichment analysis. **(E)** GO cellular component (CC) enrichment analysis.

### HIST1H4L expression in SCLC tumors

A total of 91 patients were included in this analysis, and their baseline characteristics are summarized in **Table 1**. The mean age of the cohort was 63.2 ± 7.4 years, with 75 males (82%) and 16 females. SCLC was predominantly observed in elderly males, with 53 patients over 60 years old (58%). Immunohistochemical staining of SCLC tissue specimens (**Figures 3A–C**) revealed that HIST1H4L was primarily localized in the plasma membrane and nuclei of tumor cells. In contrast, adjacent non-tumor tissues exhibited negative or low expression, with a positive rate of 8.8% (8/91). In SCLC tissues, HIST1H4L showed high expression in 53.8% of cases (49/91), indicating significantly higher expression in tumor samples compared with adjacent normal tissues. Patients were categorized into low-expression and high-expression groups based on immunohistochemical scoring. As shown in **Table 1**, HIST1H4L expression was not significantly associated with age, gender, smoking status, or clinical stage (all *P* > 0.05). The results demonstrated that patients with low HIST1H4L expression had better prognosis and higher 3-year OS compared with the high-expression group (*P* < 0.05) (**Figure 3D**), suggesting that HIST1H4L expression may be involved in the progression of SCLC.

**Table 1:** The expression of HIST1H4L was correlated with clinicopathological features in SCLC patients.

Features	Total	High expression	Low expression	*P*-value
Age(years)				0.151
≤60	34	15	19	
>60	57	34	23	
Gender				0.734
Male	75	41	34	
Female	16	8	8	
Stage				0.439
I-II	58	33	25	
III-IV	33	16	17	
Smoking				0.359
No	67	38	29	
Yes	24	11	13	
Tissues				
Tumor	91	49	42	<0.001
Normal	91	8	83	

**Figure 3:**
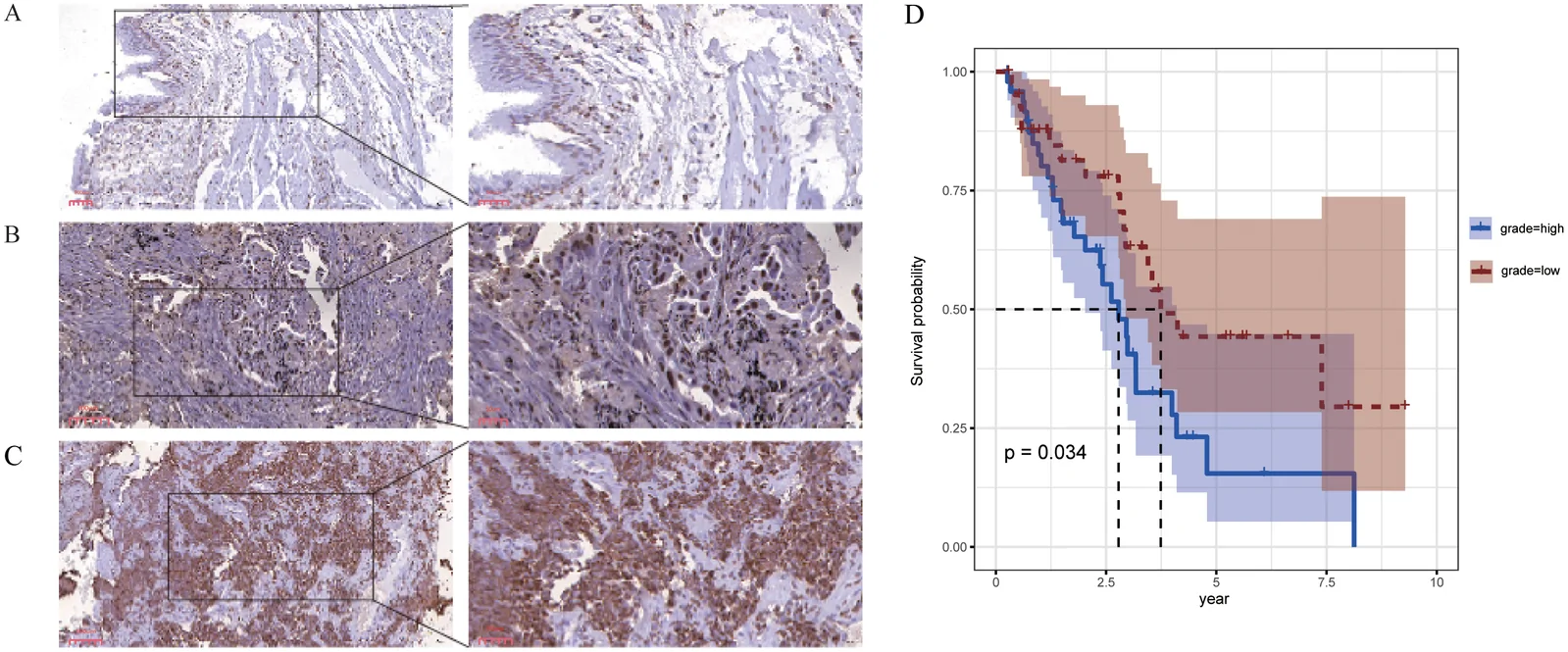
Expression of HIST1H4L in normal lung tissue and SCLC tissue. **(A)** Low HIST1H4L expression in normal lung tissue. **(B)** Low HIST1H4L expression in SCLC tissue. **(C)** High HIST1H4L expression in SCLC tissue (H&E ×200). **(D)** Association between HIST1H4L expression and prognosis in SCLC patients. (*P* < 0.05).

### HIST1H4L expression in SCLC cells and selection of the optimal shRNA

qRT-PCR was used to measure HIST1H4L mRNA levels in the normal lung epithelial cell line BEAS-2B and SCLC cell lines H446, H146, and H526. As shown in **Figure 4**, HIST1H4L mRNA was highly expressed in all SCLC cell lines compared with BEAS-2B. Among them, H146 cells showed higher HIST1H4L expression than H446 cells, and the difference was statistically significant (*P* < 0.001). H146 cells were transfected with three shRNAs targeting HIST1H4L (sh-HIST1H4L-1, sh-HIST1H4L-2, and sh-HIST1H4L-3). All three reduced HIST1H4L mRNA levels compared with the negative control, with sh-HIST1H4L-2 showing the most significant knockdown. A statistically significant difference was observed between sh-HIST1H4L-2 and sh-HIST1H4L-3 (*P* < 0.001). WB analysis (**Figure 4C**) confirmed that HIST1H4L protein levels were decreased in the sh-HIST1H4L groups compared with the control group. Therefore, sh-HIST1H4L-2 H146 cells, which showed the highest knockdown efficiency, were used for subsequent experiments.

**Figure 4:**
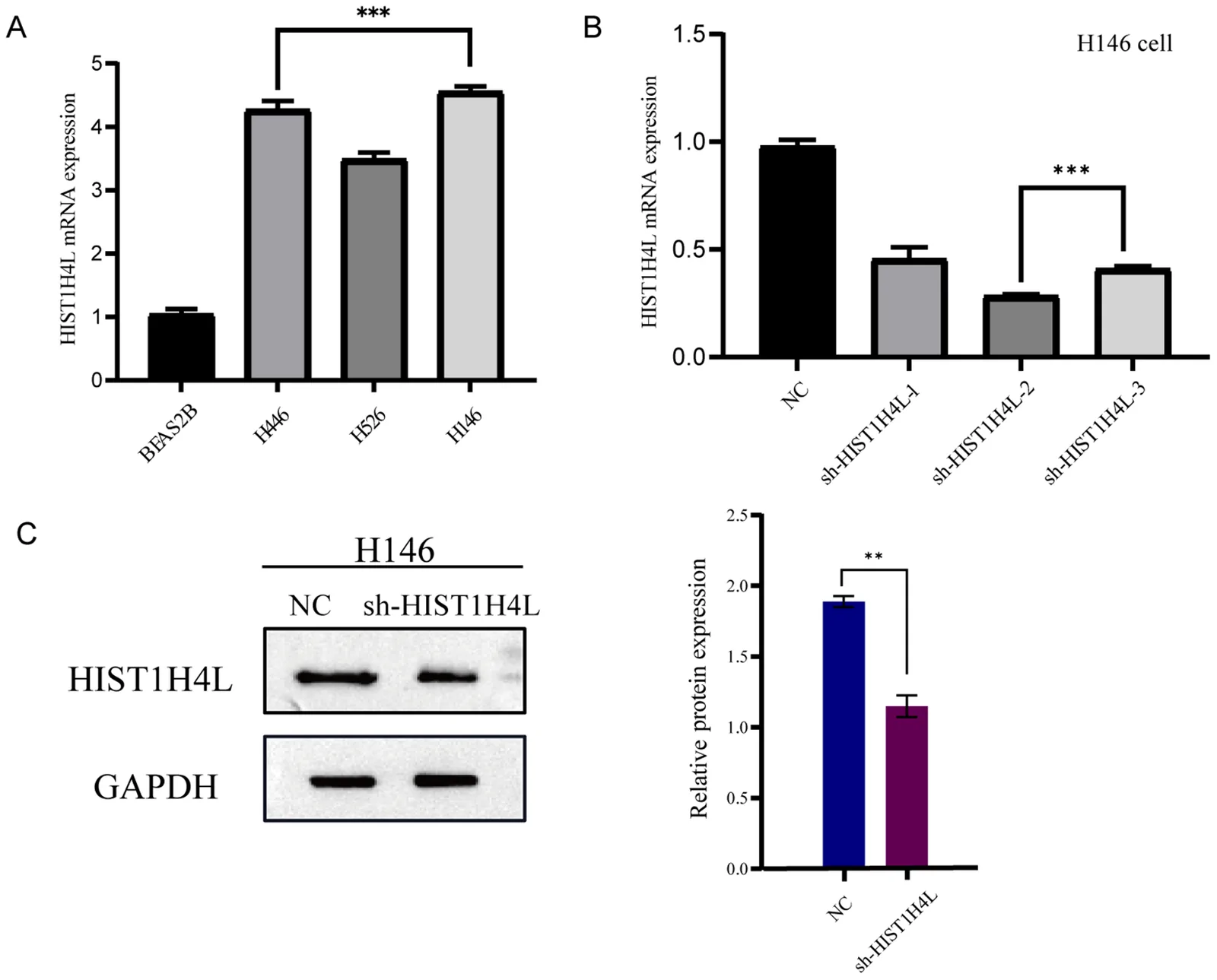
Construction and verification of HIST1H4L lentiviral transduction cell line. **(A)** qRT-PCR analysis of HIST1H4L mRNA expression. **(B)** qRT-PCR analysis of HIST1H4L mRNA. **(C)** WB showing HIST1H4L protein expression. (**P < 0.01; ***P < 0.001).

### HIST1H4L knockdown inhibits H146 cell proliferation

CCK-8 assays (**Figure 5A**) showed that HIST1H4L knockdown had no effect on H146 cell proliferation at 12 h and 24 h. However, at 48 h and 72 h, cell proliferation decreased significantly compared with the control group (*P* < 0.01). EdU assays (**Figure 4**) further demonstrated that HIST1H4L knockdown significantly reduced the number of EdU-positive cells compared with controls (*P* < 0.01) (**Figure 5B**), indicating that downregulation of HIST1H4L inhibits H146 cell proliferation.

**Figure 5:**
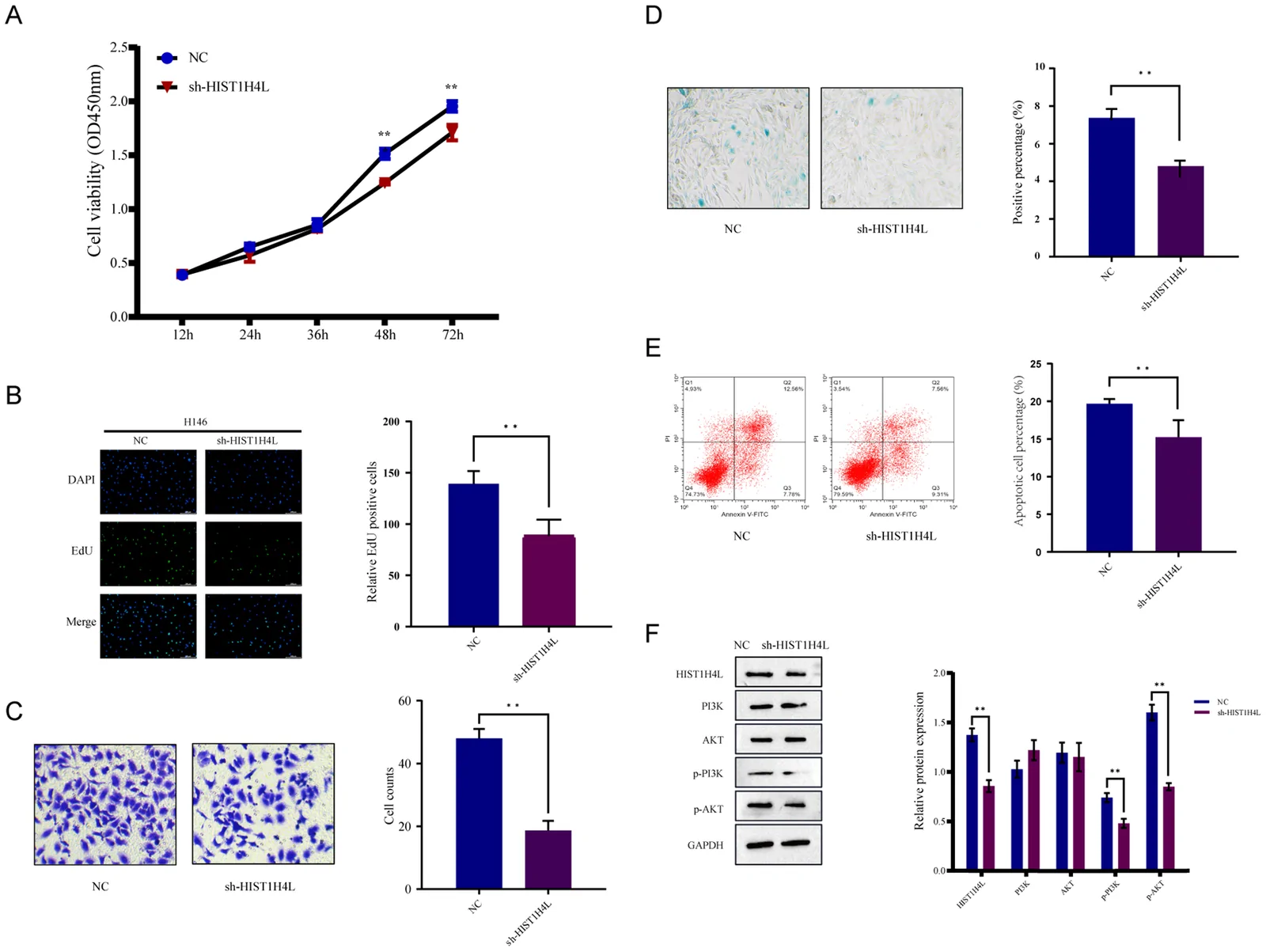
Effect of HIST1H4L knockdown on the biological behavior and signaling pathways of SCLC cells. **(A)** HIST1H4L knockdown reduced H146 cell proliferation. **(B)** Quantification of EdU-positive cells. **(C)** Invasion of H146 cells after HIST1H4L knockdown. **(D)** β-Galactosidase staining showing fewer senescent cells (blue spots) after HIST1H4L knockdown. **(E)** Apoptosis rate of H146 cells. **(F)** WB analysis showing the effect of sh-HIST1H4L on total and phosphorylated PI3K/AKT protein levels in H146 cells. (**P < 0.01).

### HIST1H4L knockdown inhibits H146 cell invasion

Transwell invasion assays showed that the number of cells passing through the membrane was significantly lower in the sh-HIST1H4L group compared with the NC group. The invasive ability decreased by approximately 60%, and the difference was statistically significant (*P* < 0.01) (**Figure 5C**).

### Effect of HIST1H4L knockdown on cellular senescence

Cellular senescence is one way the body suppresses tumors and is a potential cause of aging. β-Galactosidase staining revealed that HIST1H4L knockdown reduced the number of senescent cells (**Figure 5D**). Compared with the NC group, this decrease was statistically significant (*P* < 0.01), indicating that HIST1H4L knockdown inhibits H146 cell senescence.

### HIST1H4L knockdown promotes apoptosis in SCLC cells

Apoptosis was assessed by flow cytometry. The sh-HIST1H4L group exhibited reduced apoptosis compared with the control (**Figure 5E**). Early apoptosis rates were similar between the two groups at around 8%, but late apoptosis decreased from 12% in the control to 7% in the sh-HIST1H4L group. The reduction in apoptosis in the sh-HIST1H4L group was statistically significant (*P* < 0.01).

### Effect of HIST1H4L knockdown on the PI3K-AKT signaling pathway

To explore the mechanism by which sh-HIST1H4L affects H146 cells, we examined its impact on the PI3K/AKT signaling pathway. WB analysis (**Figure 5F**) showed that total PI3K and AKT protein levels were unchanged between the sh-HIST1H4L and control groups. However, p-PI3K and p-AKT levels were decreased in the sh-HIST1H4L group. These results suggest that HIST1H4L may promote H146 cell proliferation and invasion by activating the PI3K-AKT pathway.

## Discussion

SCLC is highly aggressive and prone to early metastasis. Its development is associated with multiple risk factors, including environmental pollution, occupational exposures, smoking, and genetic susceptibility. Clinically, SCLC initially responds well to platinum-based combination chemotherapy, showing high remission rates. However, these responses are often short-lived, and drug resistance develops rapidly, resulting in poor overall survival and prognosis (14). Compared with other cancers, SCLC carries an extremely high genomic mutation burden, approximately 7.4 protein-coding mutations per megabase (15, 16). Although exome and genome sequencing studies have been conducted, the precise molecular mechanisms underlying SCLC remain unclear. Therefore, in-depth studies of these mechanisms are crucial to identify more reliable therapeutic targets (17).

HIST1H4L is a core histone that forms part of the nucleosome and is essential for maintaining nucleosome stability. Dysregulated HIST1H4L expression can alter histone modification patterns, thereby affecting gene expression. It has been identified as a senescence-associated gene in lung adenocarcinoma (11). Based on the results obtained from GEO datasets, HIST1H4L was consistently overexpressed in multiple SCLC cohorts, with expression levels significantly higher than those in normal tissues, in agreement with previous studies (12). Protein-protein interaction network analysis indicated that genes associated with HIST1H4L were mainly enriched in transcriptional regulation and related pathways, suggesting that HIST1H4L may exert its effects in SCLC through these mechanisms.

In the present study, HIST1H4L expression was not significantly associated with patient age, sex, or clinical stage. Although clinical stage is a well-established prognostic factor, HIST1H4L expression was not significantly correlated with patient stage in this cohort, which may reflect the limited sample size and potential bias. Previous studies have reported that dysregulation of HIST1H4L may lead to selective histone modifications and abnormal gene expression, and it has been identified as a senescence-related gene in lung adenocarcinoma (7, 8). Given that the majority of SCLC patients in this study were elderly males, it is possible that HIST1H4L may influence SCLC development through senescence-related mechanisms, although experimental evidence is currently lacking.

To investigate the role of HIST1H4L in the malignant behavior of SCLC, we measured its expression in SCLC cell lines (H446, H146, H526) and normal bronchial epithelial cells using qRT-PCR. The results showed that HIST1H4L mRNA levels were significantly higher in all SCLC cell lines compared with BEAS-2B cells (*P* < 0.05). The increase of HIST1H4L protein level may be due to post transcriptional regulation rather than simple gene transcriptional level up regulation, but the specific mechanism is still unclear. Functional assays demonstrated that HIST1H4L silencing significantly inhibited SCLC cell proliferation and induced cellular senescence. Interestingly, the proportion of apoptotic cells also decreased. Transwell invasion assays revealed that HIST1H4L knockdown markedly reduced cell invasion *in vitro*. These findings indicate that HIST1H4L is a key regulator of malignant phenotypes in SCLC. By affecting processes such as proliferation, senescence, and invasion, HIST1H4L may contribute to tumor progression. It is particularly important to note that the aging process regulated by HIST1H4L in this study may be more inclined to trigger its tumorigenic aspect. The research shows that cellular senescence plays a dual role in oncology: on the one hand, it prevents the occurrence of tumors by inhibiting the proliferation of precancerous cells; on the other hand, it can promote the progression and invasion of tumors by secreting senescence-associated secretory phenotypes (18, 19).

The PI3K/AKT/mTOR signaling pathway is a central axis that regulates key physiological processes, including cell proliferation, apoptosis, angiogenesis, and metabolism. Its abnormal activation is a major driver of tumor progression, promoting malignancy by inhibiting apoptosis, accelerating the cell cycle, and enhancing angiogenesis and metastasis (20, 21). Among them, the relationship between PI3K/AKT and cell apoptosis still needs to be further studied (22). In SCLC, this pathway is also critical for cell growth, survival, and invasion, and it is closely associated with tumor phenotype changes and chemotherapy resistance (23–25). AKT kinase plays a central role in this pathway, becoming fully active upon phosphorylation. p-AKT, the active form, is highly expressed in multiple human cancers and correlates with poor prognosis (26, 27). Activated p-AKT regulates numerous downstream effectors involved in cell survival, proliferation, migration, and metabolism (28). In this study, HIST1H4L knockdown in SCLC cells significantly reduced p-PI3K and p-AKT protein levels. These results suggest that HIST1H4L may regulate key tumor behaviors, such as proliferation and apoptosis, by activating the PI3K/AKT pathway, particularly at the phosphorylation step. This finding is consistent with previous reports (29).

The inherent high heterogeneity of SCLC is a key driving factor for its acquired resistance to conventional chemotherapy (30, 31). Notably, the pronounced spatial heterogeneity of SCLC is not only a key biological feature but also provides an important basis for patient stratification and personalized treatment (32). However, a major challenge in SCLC therapy is the general lack of effective traditional drug targets, which severely limits the development of precision-targeted treatments. Therefore, identifying new molecular targets and understanding their regulatory mechanisms is crucial for advancing precision therapy in SCLC. To address this, molecular subtyping strategies based on artificial intelligence and transcriptomic analysis have emerged. These approaches classify SCLC into molecular subtypes with distinct pathway activation patterns, providing a strong foundation for the development of tailored targeted and immunotherapies (33, 34). With ongoing advances in molecular biology and drug development, targeting HIST1H4L identified in this study represents a promising therapeutic avenue, offering new hope for improving clinical outcomes in SCLC patients.

This study still has certain limitations that require further exploration in the future: Firstly, this study mainly relied on a single shRNA for gene knockdown. Although we strictly verified its knockdown efficiency, we still cannot completely rule out the interference that off-target effects may bring. Secondly, SCLC has a high degree of intratumoral heterogeneity. The single cell line model used in this study may not fully reflect the specific expression and function of HIST1H4L in different cell subpopulations. The extrapolation of the conclusion requires caution. Thirdly, this study preliminarily revealed the influence of HIST1H4L on some biological behaviors of SCLC, but the specific mechanisms of its upstream regulatory network and key downstream effect molecules have not been fully clarified. Finally, although we confirmed the regulatory effect of HIST1H4L on the PI3K/AKT pathway, this pathway in SCLC is complex and involves numerous downstream effect molecules. This study failed to conduct a systematic analysis of the key effect nodes of its downstream, and further research is needed.

## Conclusion

our study demonstrates aberrant expression of HIST1H4L in SCLC. Furthermore, we found that high HIST1H4L expression was associated with poorer outcomes in SCLC patients, suggesting that HIST1H4L may contribute to the malignant progression of SCLC. We confirmed that HIST1H4L promotes SCLC cell proliferation and inhibits apoptosis by activating the PI3K/AKT signaling pathway. Therefore, HIST1H4L as a potential molecular biomarker for SCLC but also highlights its considerable potential as a novel therapeutic target, offering new directions for improving clinical prognosis in SCLC.

## Data Availability

The datasets presented in this study can be found in online repositories. The names of the repository/repositories and accession number(s) can be found in the article/supplementary material.
